# THE INFLUENCE OF HUMOR AND SPIRITUALITY ON THE RESILIENCY OF COMMUNITY-DWELLING OLDER ADULTS

**DOI:** 10.1093/geroni/igz038.3420

**Published:** 2019-11-08

**Authors:** Bonnie L Kenaley, Zvi D Gellis, Eun hae Kim, Kimberly Mclive-Reed

**Affiliations:** 1 Boise State University, Boise, Idaho, United States; 2 University of Pennsylvania, Philadelphia, United States; 3 Texas State University, Texas, United States

## Abstract

Older adults are confronted with many distinct challenges, which require the use of various coping mechanisms to maintain psychological balance, including humor and spirituality (Bonanno et al., 2012; Koenig, 2012). This study examined the influence of humor and spirituality on resiliency of 156 (age 60 years and older) community-dwelling members of an Osher Lifelong Learning Institute located in the western region of the United States who completed a pen and paper and electronic newsletter surveys. The majority of the sample used humor to cheer themselves when feeling depressed, were amused by the absurdities of life, used humor to feel better and to cope with problems, and believed their humorous outlook prevented them from being upset or depressed. Almost three-quarters of the sample looked to a spiritual force for strength, support, and guidance, 58% worked together with a spiritual force and less than 39% thought about how their lives were part of a larger spiritual force. In the final hierarchical regression model (F (5, 143, = 8.895, p .000), only spirituality (beta = -.238, p < .001) and humor (beta = .444, p < .000) were statistically significant; whereas age, gender and living with another were not statistically significant. The findings suggests that humor along with spirituality are two internal resources that promote resiliency in older adults. Humor infused in informal interactions and planned activities as well as spiritual support may contribute to the promotion and enhancement of resiliency in community-dwelling older adults.

